# The progress of pluripotent stem cell-derived pancreatic β-cells regeneration for diabetic therapy

**DOI:** 10.3389/fendo.2022.927324

**Published:** 2022-07-28

**Authors:** Xin Wang, Mengxi Gao, Yali Wang, Yucheng Zhang

**Affiliations:** ^1^ China-Japan Union Hospital of Jilin University, Changchun, China; ^2^ The Third Norman Bethune Clinical College of Jilin University, Changchun, China; ^3^ Department of Blood Transfusion, China–Japan Union Hospital of Jilin University, Changchun, China; ^4^ Scientific Research Center, China–Japan Union Hospital of Jilin University, Changchun, China

**Keywords:** diabetes mellitus, stem cells, generation, human pluripotent stem cells, embryonic stem cells

## Abstract

Diabetes is a complex metabolic disorder of carbohydrate metabolism, characterized by high blood glucose levels either due to an absolute deficiency of insulin secretion or an ineffective response of cells to insulin, a hormone synthetized by β-cells in the pancreas. Despite the current substantial progress of new drugs and strategies to prevent and treat diabetes, we do not understand precisely the exact cause of the failure and impairment of β-cells. Therefore, there is an urgent need to find new methods to restore β-cells. In recent years, pluripotent stem cells (PSCs) such as embryonic stem cells (ESCs) and induced pluripotent stem cells (iPSC) can serve as an ideal alternative source for the pancreatic β-cells. In this review, we systematically summarize the current progress and protocols of generating pancreatic β-cells from human PSCs. Meanwhile, we also discuss some challenges and future perspectives of human PSCs treatments for diabetes.

## Introduction

Diabetes has become more prevalent in recent years. Globally, more than 463 million adults are living with diabetes. It is estimated that over 693 million adults will be affected by 2045 (1). Diabetes is mainly classified into type 1 diabetes mellitus (T1DM), which is caused by absolute lack of insulin secretion, or type 2 diabetes mellitus (T2DM), which is mostly caused by insulin resistance in peripheral organs such as the liver muscle, and fat (2). In addition to T1DM and T2DM, diabetes also includes monogenic diabetes, gestational diabetes, and latent autoimmune diabetes. The burden of diabetes is increasing all over the world, which has a severe impact on the cost of medical care and human suffering.

New drugs for the treatment of diabetes are being developed. However, current drug treatments cannot entirely prevent or reverse the progression of diabetes. Furthermore, previous studies have shown that intensive insulin therapy has the potential to improve glycemic control and alleviate diabetes but also leads to some side effects, such as hypoglycemia and weight gain. Moreover, the whole-organ pancreas and islet transplantations represent a novel method of replacing beta cells. Once islet cells are transplanted successfully, the islet cells resume releasing insulin to maintain the patient’s blood glucose levels, which will significantly improve the quality of life of diabetic patients (3, 4). However, the success of pancreas or islet transplantation as a treatment for diabetes remains limited due to the scarcity of human suitable cadaveric islet donors, variability in donor pancreas quality, and immune rejection after islet transplantation. Therefore, the urgent need is to develop an alternative source of human islet cells for diabetes patients.

More recently, research has focused on developing regenerative approaches to treat diabetes patients, and pluripotent stem cell-derived cells therapy has become a viable therapy option. PSCs, which can be classified into hESCs and iPSCs, are likely to provide unlimited supplies of pancreatic β-cells that can respond to glucose and secret sufficient insulin. Initially obtained from mammalian blastocysts over two decades ago, hESCs have the potential to differentiate into any mature cell type of the three germ layers and the capacity for extensive self-renewal (5, 6). Notwithstanding, the utilization of hESCs is primarily restricted due to ethical concerns. In contrast, iPSCs, a breakthrough in the stem cell research field, are regarded to be more promising since they can be derived from varieties of human somatic cells in patients by using several transcription factors (7–9), thereby creating patient-specific cells that perfectly circumvent immune rejection after implantation, while resembling ESCs morphologically and functionally. Totally, the use of PSCs has generated significant outcomes in cell replacement therapy, drug screening, and disease modeling.

Tremendous progress has been made in generating glucose-responsive human stem cell-derived β-cells, which is an emerging promising therapeutic approach for diabetic patients. In this review, we summarize advances in the generation of pancreatic β-cells from human pluripotent stem cells, the differentiation of hESC/iPSC-derived cells into pancreatic β cells and islet-like organoids, and discuss the limitations and challenges for their successful generation and therapeutic application in diabetes.

## Development of protocols for differentiation of PSCs into pancreatic β-cells

**Figure 1:**
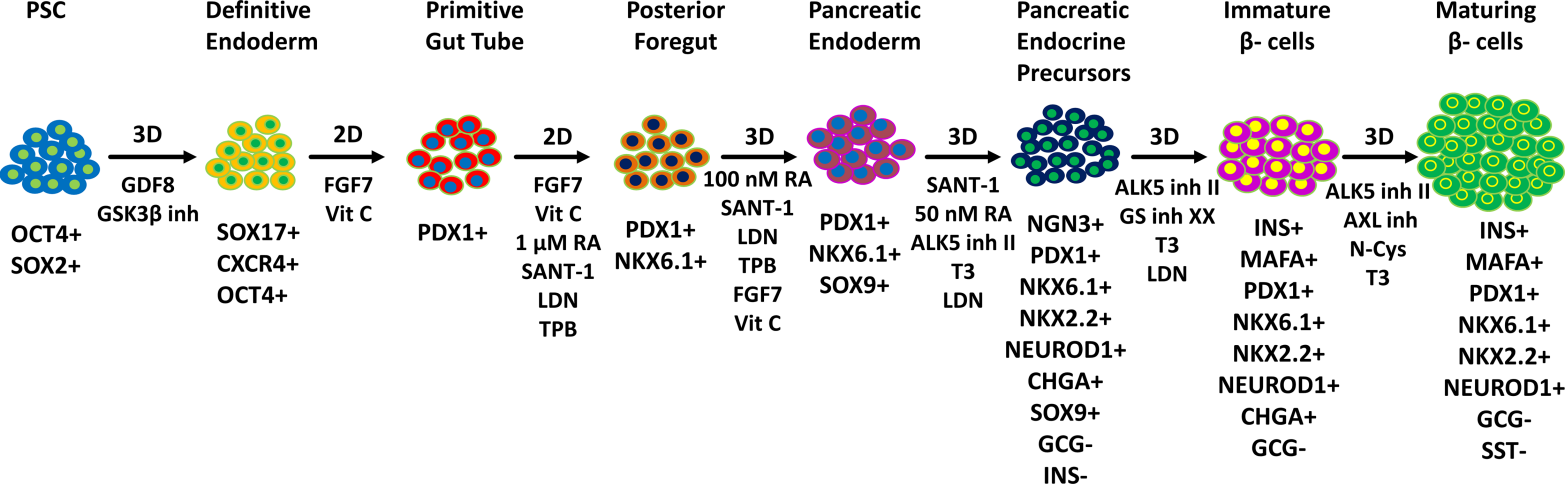
Differentiation protocols of pancreatic β-cells from human iPSCs. Through mimicking the normal human pancreas development, directed differentiation protocols *via* adding different growth factors and small molecules can induce human iPSCs into pancreatic β-cells *in vitro*. This figure summarizes the 7-stage differentiation protocol and key markers of the differentiation pancreatic cells for evaluation of the consecutive stages of differentiation [(mainly based on references (20, 21)].

The protocols for differentiation of PSCs into pancreatic β-cells are designed to mimic normal pancreatic development by sequential treatment of PSCs with various combinations of growth and signaling molecules added to the culture medium (10–13). Recent progress has crystallized in differentiation protocols that make possible the efficient differentiation of PSCs into pancreatic β-cells. See **Table 1** and **Figure 1** for an overview of the protocols for the generation of stem cell-derived beta cells.

**Table 1:** Overview of protocols for the generation of stem cell-derived β-cells.

Sources	Cultures	Improvements	Outcomes and significance	Reference
hESCs	Planar culture and air-liquid interface	-Supply of Vitamin C to downregulate NGN3 during S2-4 -Transferring to an air-liquid interface plus the addition of ALK5iII and T3 during S5 -The combination of ALK5iII, T3, LDN and GSiXX during S6 -Addition of N-Cys during S7	**-**Elevated expression of key markers of mature pancreatic β-cells such as MAFA -Ameliorating hyperglycemia in diabetic mice within 40 days -Establishment of a standardized and classical 7-stage protocol for the generation of β-like cells	(14)
hESCs and iPSCs	Suspension culture	**-**Extension of time during PP2 with KGF, SANT1 and retinoic acid -A distinct combination of 11 factors and molecules including PdbU to differentiate PPs	**-**Similar calcium flux in response to glucose **-**Secreting quantities of insulin comparable to adult β cells in response to multiple sequential glucose challenges *in vitro* -Higher proportion of mice that survived when transplanted with SC-β cells within 8 weeks	(15)
hESCs and iPSCs	Suspension culture	-Regulation of Alk5i to inhibit or permit TGF-β signaling pathway in S6 -Use of ESFM in S6 to create a serum-free protocol	-Increased insulin secretion with glucose rising -Mostly expressed CHGA and C-peptide -Improved β cell markers expression -Improved glucose tolerance *in vivo* -Rapidly controlling glucose in STZ mice within 10 days -Functional improvements compared with previous SC-β cells	(16)
hESCs	Suspension culture	**-**Use of the INS^GFP/W^ cell line to optimize previous protocol **-**Addition of a re-aggregation step for the cluster of immature β cells -Co-localization with TOMM20 and measuring rhodamine-123 to reveal the mechanisms of mitochondrial function to the maturity of β cells	-Mostly expressed key markers of β cells and C-peptide -Improved maturation of target cells **-**Calcium flux in response to glucose **-**Sulfonylurea reaction	(17)
hESCs	3D culture	**-**Re-aggregation technique to deplete non-endocrine cells **-**Identifying CD49a (also known as ITGA1) as a surface marker of the β-cell population -Use of magnetic microbeads with anti-CD49a for efficient sorting of SC-β-cells	**-**Stabilized glucose responsiveness **-**Generation of high purity SC-β-cell clusters -Revelation a CD49a as a surface marker for enriching β cells *in vitro* -As a reference for future studies on the differentiation of β-cells	(18)
iPSCs	Gellan-gum-based 3D culture	-Inclusion of hADSCs and HUVECs in the formation of multicellular spheroids (MCSs) from PPs -Addition of WNT4 in the functional maturation process of HILOs	-Improved oxidative metabolism and *in vitro* GSIS -Ameliorating hyperglycemia in mice and maintaining normoglycemia for more than 6 weeks -Identifying the non-canonical WNT signaling as a necessity to the metabolic maturation of HILOs	(19)
hESCs and iPSCs	Suspension and 2D culture	-Dispersing spheroids into single cells and interacting with basement membrane proteins such as laminin 511 for 3 days on monolayer culture	-Enforced β-like cell polarity -Reduced basal insulin secretion and increased stimulated insulin secretion -Uncovering the role of basement membrane proteins during differentiation and maturation *via* cell polarization	(20)
hESCs and iPSCs	Planar culture	-Treatment with 1 μM latrunculin A for the first 24h at S5 -Elimination of the need for suspension culture	-Similar expression of pancreatic β-cell markers including MAFB and a smaller proportion of somatostatin and glucagon positive cells -Similar insulin content and increased biphasic GSIS -Similar rates of reconstructing normoglycemia in STZ-induced mice and maintaining the state for at least 9 months -Compatible to various cell lines -Revelation of the connection between actin cytoskeleton and the differentiation of pancreatic cells	(21)
hESCs	V-bottom plate and air-liquid interface	-Insertion of GFP and NLS into NKX6.1 gene -Use of V-bottom plate to promote the 3D structure aggregation of PPS -A combination of ten chemicals (PP-10C) to maintain NKX6.1 expression and 3D structure -Three combinations of chemicals and factors for the stepwise induction of β-cells -Late-stage readout strategy in the screening process	**-**Higher efficiency for the generation β-cells from multiple cell lines and enrichment for the desired cell types -Similar C-peptide level and insulin secretion in response to high-concentration glucose -Restoring normoglycemia in STZ-induced mice within ~2 weeks	(22)
hESCs and iPSCs	Planar culture	**-**Treatment with latrunculin A at the start of S5, with dosage and timing depending on different cell lines -Elimination of the need for suspension culture	**-**Establishment of a six-stage planar differentiation protocol with expanded accessibility and simplified and economic generation procedures -Robust production of functional β-like cells with double or triple the number of cells per volume compared to suspension protocols, displaying static and dynamic GSIS -Compatible to various cell lines	(23)
hESCs and iPSCs	Planar culture or suspension culture	-Replacing Activin A with dorsomorphin (DM) -RPMI basal medium with HAS plus Vc	-Increased population of DE with up to 87% efficiency of SOX17 and FOXA2 double positive -Enhanced cell survival and decreased costs -Establishment of a xeno-free “GiBi” protocol for the generation of PPs which possess the capability of differentiating into pancreatic lineages -More insulin secretion in response to high-level glucose -Identifying the inhibition of BMP signaling to improve the efficiency of DE induction	(24)
hESCs and iPSCs	Monolayer, microwells, and suspension culture	**-**Addition of nicotinamide, epidermal growth factor, Activin A and ROCKi during S4 -Aggregation during S5 in microwells -Maturation in suspension culture replacing ALK5i with ZM447439, T3, and NAC	-Completion of a comprehensive analysis of the functional maturation process *in vitro* and *in vivo*, including lower proportion of undesired cell types, downregulation of markers related to proliferation, reorganization of cytoarchitecture, increased insulin content, and analogous biphasic GSIS.	(25)
hESCs	Basal and suspension culture	**-**Coculturing mesenchymal and endothelial cells (M-E cells) with PPs for a week -Addition of WNT5A to EPs for 3 days	-Increased number of insulin positive and C-peptide positive cells -Sensitive insulin and C-peptide secretion in response to high-concentration glucose and KCl *in vitro* -Discovery of WNT5A as an inducer to promote differentiation and maturation of pancreatic β-cells by activation of non-canonical WNT5A/JNK signaling and inhibition of BMP signaling	(26)
hESC	Matrigel 3D culture and microwell chips	-Coculturing PPs with hFP-MC at a ratio of 1:1 in microwell chips for 2 days and transferring to static suspension culture for 4 days to produce DC-PA	-Upregulation of NGN3 and INSULIN genes -Consistent sized, spheroid-like structure and stability of DC-PA -Proposing a scalable strategy to produce islet organoids	(27)
hESCs and iPSCs	3D culture	-7-days treatment of the bromodomain and extraterminal domain (BET) inhibitor I-BET151 -Use of EF6I (the combination of EF6 medium and I-BET151) medium	**-**Promoting the expansion of PPs -Revelation of the molecular mechanisms of I-BET151 -Lowering significantly glucose within 2 weeks and maintain normal levels after 3 weeks in STZ-induced mice	(28)

### Before 2014

The initial discovery of spontaneous differentiation of hESCs into insulin-secreting cells (IPCs) that express vital genes, GLUT2 and islet-specific GK, *in vitro* has opened the possibility for directly differentiating PSCs into pancreatic β-cells in response to glucose for cell-based therapy in diabetes mellitus (29). Following the differentiation of embryoid bodies (EB) from hESCs, when growing the cells in suspension culture to promote the formation and maintenance of EB, the spontaneous process occurs much more frequently. The size of cell clusters and the addition of mesoderm-derived embryonic cells also play an essential role in the differentiation process (30). However, the transcriptional factors underlying this phenomenon are unknown, and the efficiency of the spontaneous process is low, which prompts the establishment of a standardized and productive protocol for transplantation therapy.

PSCs must undergo some key phases to mature into β-cells which include the formation of definite endoderm (DE), primitive gut tube, posterior foregut, pancreatic progenitors (PPs), and finally, hormone-expressing endocrine cell (ECs). The first step towards DE was accomplished in early studies with the use of activin A and decreased serum. Cell cultures containing ~80% SOX17 and FOXA2 dual positive definitive endoderm cells were successfully produced (31). A five-stage protocol was established by the same group the following year that recapitulates the pancreatic development *in vivo* (32). It is a milestone in the progress toward generating mature β-cells. Although an average of 7% of insulin-expressing cells are detected in the final product, more than one hormone is expressed in one single cell. In other words, most of them are polyhormonal instead of monohormonal, analogous to immature pancreatic ECs (33). Another evidence of the immaturity of the product is poor responsiveness to glucose, while glucose-stimulated insulin secretion (GSIS) is considered an important feature in mature β-cells. Besides, the expression of two key transcriptional factors, NKX6-1 and PDX1, cannot be observed in the final product (32). Later, improvements were made to this protocol, and ECs that exhibit GSIS were obtained after implanting stage-4 cells, namely pancreatic endoderm, into mice. The concentration of insulin stimulated by glucose resembles the control group in which nearly 3,000 human islets of Langerhans were transplanted. More strikingly, these cells are shown to ameliorate hyperglycemia and reverse diabetes in streptozotocin (STZ)-induced mice with considerable co-expression of NKX6-1 and PDX1 (34).

### After 2014

In 2014, a seven-stage protocol was built which successfully enabled the generation of β-cells that are monohormonal and possess the property of MAFA expression induced by the combination of R428, ALK5 inhibitor, and T3 in the last stage of the differentiation process *in vitro*, although delayed insulin release in response to glucose was observed due to a slower Ca^2+^ response (14). In the same year, another group proposed a protocol that also reached the goal of producing monohormonal β-cells that display GSIS in a 3D culture system, despite the differences in gene expression patterns from mature pancreatic islet cells (15). Both resulting cells are able to ameliorate hyperglycemia and reverse diabetes in STZ-induced mice within a short period, paving the way for follow-up studies and cell replacement therapy for patients suffering from diabetes. The protocol was further simplified by discarding the use of BMP inhibitors, thereby avoiding precocious endocrine induction with subsequent exposure to retinoic acid and EGF/KGF (35). Insulin-producing cells can also be obtained from human fibroblasts (36) and even patients with T1DM (37), resembling those from non-diabetic patients.

Since then, significant efforts have been made to optimize the protocol to enhance the efficiency of the generation of β-cells with a closer resemblance to *bona fide* pancreatic β-cells.

#### Resizing and reaggregation

By modulating TGF-β signaling pathways, the differentiation into β-cells exhibiting GSIS is achieved successfully, indicating the critical role TGF-β plays in the differentiation process. It is shown that its blocking during stage 5 can improve the efficiency but hamper GSIS during stage 6. Notably, resizing the clusters in stage 6, which can decrease the average diameter by more than 50%, and an enriched serum-free media culture in this strategy also promote differentiation significantly (16). Another improvement to the differentiation protocol is to include a clustering/reaggregation step of immature β-cells *in vitro* to form enriched β-clusters which exhibit improved functional characteristics and key cell markers. The clustering/reaggregation process is reported to lead to enrichment in oxidative metabolic pathways and ERRγ, thereby maturing mitochondrial metabolism. Although a small proportion of dual-hormone-positive cells are also detected in addition to β-cells, tumors are not found 8 months after transplantation, indicating the increased safety of this approach (17). By utilizing such a reaggregation step, Liu et al. devised a protocol that efficiently produces β-cell islets from several pluripotent stem cell lines with a higher concentration of desired cell types. Remarkably, their guiding principles, including proper prolongation of specific differentiation steps to ensure the full acquisition of each cell state, and the employment of the late-stage gene markers referred to as the late-stage readout strategy, have successfully led to more promising outcomes (22).

#### Metabolic functionality

In addition to the focus on constructing the mature structure of β-cells from iPSCs, scientists also attempt to illuminate their mature metabolic functionality. The metabolic immaturity of stem cell-derived islets includes 1) failure to respond to glucose in mitochondria; 2) reduced level of glucose-6-phosphate, 3-phosphoglycerate, phosphoenolpyruvate, and lactate, namely a bottleneck in glycolysis; 3) discrepant production of TCA-originated metabolites and redox shuttle components (25, 38). The induction of ERRγ can promote the activation of oxidative phosphorylation in mitochondria, thereby enhancing the production of ATP, which is indispensable for pancreatic β-cells to maintain high energy levels and secret insulin stimulated by glucose (39). It is reported that the overexpression of non-canonical WNT4 signaling can upregulate mitochondria-related genes through the ERRγ gene networks. In this study, iPSCs-derived pancreatic islets treated with WNT4 in the final maturation step have similar transcriptional patterns with human primary pancreatic islets, including key islet cell markers and, notably, ESRRG encoding ERRγ. The resulting islets show robust insulin secretion, highlighting the necessity of mature mitochondria in the regulation of GSIS in β-cells (19). Remarkably, based on an optimized protocol, Balboa et al. uncovered the functional mechanisms underlying the continuous maturation process by a series of analyses and assays, reporting the heterogenous maturation trajectory and the immaturity of the products *in vitro.* Compared to native pancreatic islets, SC-islets showed a reduced mitochondria TCA metabolite accumulation, lowered ATP/ADP ratio, and the disappearance of respiration spikes. Furthermore, the upregulation of MAFA and RBP4, markers for islet maturation, occurs 3 ~ 6 months post-transplantation (25).

#### Microenvironment

The islet of Langerhans includes α-, β-, δ-, ϵ- and pancreatic polypeptide cells, which regulates the glucose level as a whole. For example, glucagon secreted by α-cells can increase the glucose concentration and regulate insulin secretion (13). The microenvironment surrounding the β-cells is vital to insulin secretion and maturation, including the cell-to-cell interaction and cell-to-matrix interaction (40). Thus, it is of paramount importance to understand and mimic the microenvironment to obtain fully mature β-cells that exhibit GSIS.

Extracellular matrix (ECM) proteins such as laminin and collagen can downregulate integrin α5 and thus induce endocrinogenesis. The contact of ECM with integrin α5 directs the PPs to ductal cells through the F-actin–YAP1–Notch mechanosignaling axis, while its inhibition can also promote the differentiation towards endocrine cells. It is shown that when exposed to a siRNA against ITGA5 or antibodies against integrin α5, NGN3, INS, and GCG are upregulated, indicating the functional maturation of hormone-expressing cells (41). The interaction with basement membrane proteins among all the ECM proteins is critical in activating integrin, forming focal adhesions, and thus maintaining the 3D structure of β-cells (42–44). Basement membrane proteins are found in the differentiated spheroids in a diffuse web-like network as a “by-product” with co-expression of fibroblast genes. In primary human islets, basement membrane proteins are secreted by vascular endothelial cells and originated from mesodermal lineage. However, the expression of CD31 and CD34, two endothelial markers, cannot be detected by a bioinformatic analysis and immunostaining in differentiated spheroids. It still remains elusive where basement membrane proteins in the spheroids come from, and further research needs to be done (18, 20). Despite the expression of polarity determinants and presynaptic proteins in β-cells within spheroids, they are disorganized, leading to unclear polarization of β-cells, while an organized polarity plays a vital role in the functionality of β-cells (45). Basement membrane proteins interact with the basal region, one of the three domains facing the capillaries, enriching presynaptic scaffold proteins which regulate GSIS (46, 47). Notably, if cultured on basement membrane proteins, the stimulation index is increased from 2.8 to 3.9-fold in spheroids containing PSCs-derived β-cells. Moreover, clear polarization and apparent segregation between the apical and basal poles can be detected in β-cells exposed to basement membrane proteins, suggesting the maturity of these β-cells derived from hESCs or iPSCs. By establishing ECM analogous to the native islet of Langerhans and the use of basement membrane proteins, the goal of generating mature β-cells for clinical use in regenerative medicine is coming closer (20).

The microenvironment in the pancreas offers key growth factors and signals to endocrine cells during their differentiation and maturing process (48), which are not fully elucidated. According to different phases of the process, the microenvironment presents different characteristics dynamically, mesenchyme and endothelium cells included (49, 50). Mesenchyme and endothelium cells (M-E cells) obtained from various endodermal organs and stages and divided into several groups (termed from M-E9 to M-E20) were co-cultured with hESC-derived PPs. It was revealed that M-E17 and M-E20 cells secreted an increased amount of C-peptide by 8.6~9.3 and 2.2~2.4-fold, respectively, compared to the control groups. Further investigation into the molecular mechanisms identified WNT5A as an initial activator through the non-canonical (JNK/c-JUN) WNT signaling pathway (26). Moreover, Ghezelayagh et al. also co-cultured human fetal pancreatic-derived mesenchymal cells (hFP-MCs) with hESC-derived PPs and discovered the upregulation of insulin secretion in co-cultured differentiated β-cell spheroids (27).

#### Cytoskeleton

Integrins are transmembrane proteins that mediate the communication between ECM and actin cytoskeleton, which allow the movement and the possibility of changing the shape of cells. *Via* the role of integrins, ECM can regulate the recruitment of adhesion proteins and cytoskeletal polymerization and modulate the downstream signaling pathways, thereby influencing the differentiation direction of PSCs (21, 51–54). Recently, it was reported that NEUROG3 expression levels in PPs can be lowered by a polymerized cytoskeleton, inhibiting specification towards mature β-cells, which can be blocked by 24-hour treatment with latrunculin A, in conjunction with adequately timed cytoskeletal depolymerization. Previous studies favor the use of 3D culture to reconstruct the microenvironment surrounding β-cells to promote cell-to-cell and cell-to-matrix interaction. However, in this study, a planar culture protocol was applied to four cell lines, and the result was surprising. The resulting β-cells display dynamic GSIS and ameliorate diabetes in mice faster than those obtained in suspension cultures. It is even more surprising that cytoskeletal states can determine cell fate commitment in a wider range (21). Noteworthily, based on the theory mentioned above, this group proposed a protocol entailing the use of cytoskeletal depolymerizing compound latrunculin A completely on a planar culture (tissue culture polystyrene), which simplified the pancreatic differentiation process and made possible the scalable manufacture of stem cell-derived β-cells (23).

#### Small molecules

Besides the classical strategies for inducing mature β-cells, which commonly involve the modulation of key signaling pathways that determine β-cell specification, such as PI3K, FGF, BMP, WNT4, SIX2, and TGF-β (16, 19, 32, 55), small molecules are also of significant importance not only in the reprogramming of iPSCs but in the conversion of PSCs to mature β-cells. IDE1 and IDE2 were first identified to induce 70-80% of mouse ESCs into DE after screening 4,000 compounds (56). Follow-up studies discovered the capacity of (-)-indolactam V (ILV) to direct the induction of PDX1-expressing PPs from DE together with FGF10, probably due to the activation of protein kinase C (PKC) signaling (57). Forskolin, dexamethasone, and a TGF-β inhibitor are used as inducers to convert PPs to IPCs (58). Recently, a group found a compound, sodium cromoglicate (SCG), which can improve the differentiation efficiency by approximately 10% based on a refined version of a previous protocol (59). More recently, Liu et al. found that PPs treated with a combination consisting of ten chemical compounds, namely “PP-10C” after testing 21 combinations, can maintain the expression level of NKX6.1, a crucial marker for the production of monohormonal β-cells. Further differentiation into β-cells with increased efficiency requires the use of a combination of eight chemicals/factors (termed as “EP-8C”) at stage 6, a combination of nine chemicals/factors (termed as iβ-9C) at stage 7, and a combination of seven chemicals/factors (termed as Fβ-7C) at stage 8. These four cocktails of chemicals and/or factors support the generation of β-cells with higher yield. See **Table 2** for a detailed description of the chemicals and factors in this protocol.

**Table 2:** A detailed description of the chemicals and factors in the protocol.

Name	Contents
PP-10C	LDN (an inhibitor of BMP signaling) T3 (thyroid hormone), RA, EGF SANT1 (an inhibitor of sonic hedgehog signaling), Repsox (an ALK5 inhibitor) ZnSO_4_,(2S,5S)-(E,E)-8-(5-(4-(trifluoromethyl)phenyl)-2,4-pentadienoylamino) benzolactam (TPB, a protein kinase C activator) NICO (a form of vitamin B_3_) γ-aminobutyric acid (GABA, a neurotransmitter)
EP-8C	FSK (a cAMP pathway activator) Repsox (only 1 µM) LDN, TPB, KGF, SANT1, RA, T3
iβ-9C	LDN, T3, Repsox, ZnSO_4_ GSIXX (a notch pathway inhibitor) RA, HGF, IGF1 PD173074 (PD, a fibroblast growth factor (FGF) pathway inhibitor)
Fβ-7C	betacellulin (BTC) ISX-9 (a NEUROD1 inducer) G-1 (a G protein-coupled estrogen receptor agonist) Deza (a histone methyltransferase inhibitor) ZM (an aurora kinase inhibitor) H1152 (a ROCK-II inhibitor) CI-1033 (a pan-ErbB inhibitor)

Mainstream studies commonly involve the use of Activin A to promote DE formation (15, 60). However Jiang et al. abandoned Activin A during DE stage and instead replaced it with dorsomorphin, an inhibitor of the BMP ALK2 receptor. Dorsomorphin, combined with CH, enhanced the number of the resulting DE that co-expressed SOX17 and FOXA2. Remarkably, DE obtained from this xeno-free strategy (termed as GiBi protocol) could be further induced into PPs and, finally pancreatic β-cells that displayed dynamic GSIS (24). Since this small molecule-based GiBi protocol omits high-cost Activin A, the costs decrease dramatically by 99% compared to published Activin A-involved protocols while tackling the problem of massive cell death (61), thereby paving the way for the clinical applications of cell-based therapy for diabetes. Meanwhile, by adding I-BET151 to inhibit bromodomain and extraterminal domain (BET), PPs with PDX1 and NKX6.1 dual positive can be maintained and expanded, providing novel supplies for regenerative research and transplantation therapy which is restricted by limited cadaveric donors (28).

#### The application of scRNA-seq

Single-cell RNA sequencing (scRNA-seq) can analyze the transcriptional profiles of individual cells using a microfluidic droplet platform, thereby identifying the specific cell types of the PSCs-derived β-cells (62). This technology can unveil the heterogeneity of the cell clusters derived from the differentiation of PSCs and has been applied to various fields to gain a comprehensive view of β-cell differentiation (18, 55, 63–66).

Four types of cells were identified in the products of β-cell induction from iPSCs, including stem-cell-derived β (SC-β) cells co-expressing INS, NKX6.1, ISL1, and other β-cell markers, and three other non-β-cells (18). Polyhormonal stem-cell-derived α (SC-α) cells expressing GCG, ARX, IRX2, and also INS were characterized as α precursors in human fetal pancreatic development. A population of endocrine cells (SC-EC) similar to enterochromaffin cells, which serve as chemosensors in the intestine (67), co-express several marker genes related to enterochromaffin and the synthesis of serotonin such as TPH1, LMX1A, and SLC18A1. Surprisingly, a cluster of non-endocrine cells was observed with the absence of key endocrine markers and differentiated into the pancreatic exocrine cells (18). More importantly, the traits of GSIS and global transcriptional profiles were preserved in later studies, indicating that these traits do not require exogenous factors or serum (18). Of note, another team compared the gene expression profiles of PPs generated from PSCs based on three previously published protocols (14, 35, 66, 68). PPs generated based on two protocols (14, 68) resembled their counterparts obtained from human fetal pancreas. Therefore, scRNA-seq is promising in elucidating the molecular mechanisms underlying the differentiation and maturation of β-cells.

## Generation of pancreatic islet organoids for diabetes therapy

**Figure 2:**
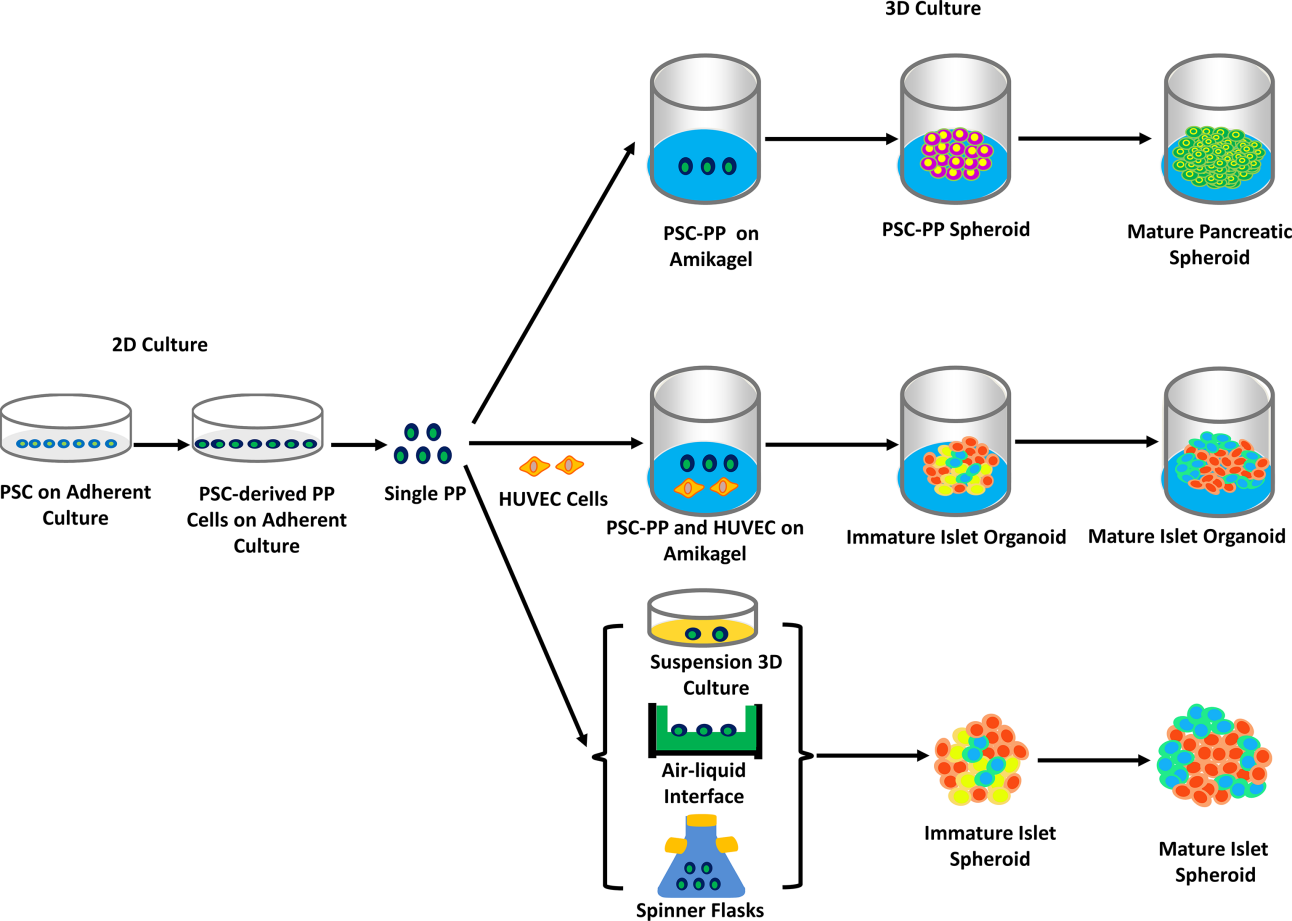
The generation of hPSC-derived islet spheroids and organoids on 2D or/and 3D culture. PSCs were grown, differentiated, and eventually turned into pancreatic precursor cells (PSCs-derived PP cells) in adherent culture. PSCs-derived PP cells were seeded to amikagel to form mature spheroids, or together with HUVEC cells to form islet-like organoids. Other methods, such as PSCs-derived PP cells placing in the gas-liquid surface or spinner culture flasks, form islet spheroids (mainly based on references 20, 21, 74 and 75). Abbreviation. Human endothelial cell, HUVEC.

From the very beginning of generating pancreatic β-cells, much attention has been paid to converting iPSCs into functionally mature β-cells that display GSIS. A recent study showed that the morphology of the cell clusters had a significant impact on their differentiation into highly mature β-cells (69). Pancreatic islet organoids derived from PSCs and adult stem cells consist of all types of islet cells with similar 3D structures and functionality to native pancreatic islets. The generation of β-cells, which has been reviewed above, is at the core of islet organoids (see **Figure 2**). However, of significant importance is the generation of other islet cells, hormone-expressing, and non-hormone-expressing cells included, which altogether create an appropriate microenvironment for the survival and function of IPCs. Transplanting organoids provides protection to support the functionality of pancreatic β-cells, while mere β-cells can hardly survive or display efficient functions since they are normally transplanted into other sites instead of pancreatic islets. Such organoids provide an ideal disease model for the investigation into diabetes therapy and drug screening.

In 2016, it was among the first to report that dissociated hormone-expressing cells differentiated from ESCs have the capacity to cluster spontaneously to form endocrine cell clusters (ECCs) in one day, with their size varying from 50~150 μm, analogous to native human islets whose diameter are approximately 100 μm. ECCs, containing several types of endocrine cells with α-cells as an exception which is an imperfection of the ECCs in this study, perform robust secretion of insulin and C-peptide, however, in response to stimuli including high-level glucose and the inhibition of potassium channel. On stimulation by glucose, an instant Ca^2+^ influx is detected, and insulin granules are observed in the cytoplasm of ECCs, indicating the rapid response of ECCs *via* Ca^2+^ oscillation. Moreover, hyperglycemia in STZ-induced mice suffering from diabetes is ameliorated within 3 days after transplantation with ECCs, faster than those with PSCs-derived endocrine cells, despite the low survival rate after day 12 (70). A revolutionary strategy involving an organ-on-a-chip system based on 3D suspension cultures was devised to produce islet organoids *in situ*. A perfusable multi-layer microfluidic chip composed of four parts that can accurately control the components of ECM, cell-to-matrix interaction, and circulatory flow was applied to the differentiation process of iPSCs (71). Dissociated iPSCs were injected into the top layer of the chip, followed by the addition of several growth factors known to induce the differentiation process efficiently. It was shown that the formed organoids tended to maintain their spherical shape and have smooth and closely connected surfaces with the upregulation of E-cadherin, a cell junction protein that regulates the formation of 3D structures in native islets, PDX1, and NKX6.1. Besides, the organoids cultured in the chips were found to react to glucose more sensitively and secrete a higher insulin level, due to a more immediate Ca^2+^ influx than static models.

Moreover, collagen-based scaffolds, which offer ECM compositions and allow the formation of 3D structures of iPSCs, with the combination of Matrigel consisting of several basement membrane proteins, provide a better alternative for the generation of islet organoids (C-M scaffolds) (72). An increase in the levels of the expression of pancreatic marker genes, including Sox17, Pdx1, Ngn3, Insulin, Glut2, and MAFA, was observed in the generated islet organoids in C-M scaffolds compared to 2D conditions. Notably, most insulin-producing cells were monohormonal which means somatostatin, glucagon, or PP were not expressed, and a 12-fold increase of insulin secretory granules was detected. These are indicators of the maturity of pancreatic β-cells. A hydrogel platform named Amikagel enabling the robust formation of islet organoids of precise size and cellular heterogeneity (73), and later a hydrogel system used for clustering islet cells originated from hESCs with endothelial cells (74), and recently a continuous microfluidic system that involved an array of droplets in generating hybrid hydrogel capsules containing islet organoids (75), all produced islet organoids with highly similar morphology and functionality to primary pancreatic islets and high level of the expression of β-cell markers. Cells cultured in microporous scaffolds also exhibit better GSIS by accurately modulating the size of the cell clusters at 250~425 µm (76). The perfusable organ-on-a-chip platform, which has been discussed above, also contains a through-hole PDMS layer and a polycarbonate porous membrane in the middle (71). Recently, Tao et al. proposed an innovative multi-organoid-on-chip system co-culturing liver and pancreatic islets, the two crucial organs in modulating circulation glucose levels and maintaining normoglycemia, for up to 30 days from iPSCs. The co-cultured organoids can improve hepatic glucose uptake and display more sensitive GSIS, which is not observed in mono-organoid culture. This new system provides a source for the research and therapy of T2DM (77). Consequently, transplanting islet organoids instead of pure β-cells into recipients is regarded as a more reasonable and appropriate strategy for treating patients suffering from diabetes.

## On reaching the ultimate goal: Latest breakthrough on stem-cell based clinical trials

Remarkably, Ramzy et al. and Shapiro et al. published their preliminary results from an ongoing phase I/II clinical trial using a device (VC-02) that allows the entry of capillaries launched by ViaCyte (NCT03163511), which is a pioneer in the field of transforming the success of utilizing PSCs-derived pancreatic islet cells to ameliorate hyperglycemia on diabetic animal models to T1DM patients (78, 79). The clinical trial was initiated in 2017 and was estimated to complete by November 2023 in which pancreatic endoderm cells (PEC-01) encapsulated within a device that permits vascular growth and promotes cell survival are transplanted subcutaneously, while C-peptide level is considered as a main indicator of the outcomes of the trial (80, 81). Thus, the use of immunosuppression becomes a necessity to avoid the attack of immune cells on transplanted sites since this encapsulation device cannot isolate PEC-01 from the hosts’ immune system.

In Shapiro’s multicenter study, 17 patients were enrolled, including 9 males and 8 females who had all suffered from T1DM for at least 8 years. The outcomes showed that 6 participants displayed increased C-peptide levels within 6 months at the earliest, and by immunohistochemical staining of the explants removed from the patients at certain points during the trial, the number of insulin-positive endocrine cells in the explants from responders (Circulation C-peptide > 0.1 ng/mL) is significantly higher than non-responders. Meanwhile, the safety and tolerability of VC-02 are also well tested in the trial, which reported participants with only mild adverse effects mostly correlated with surgical procedures and immunosuppressive drugs (79). Ramzy et al. further confirmed that the rise of C-peptide levels could be attributed to the implants due to the loss of meal-stimulated C-peptide in two patients after the removal of the implants and the existence of mature MAFA positive β-cells. In their trial, among the initial 15 participants (7 males and 8 females) from a single center, almost all exhibited increased C-peptide level 26 weeks post-implantation, while 4 patients reached 30 pM. In addition to side effects unassociated with implanted PEC-01, teratoma is also not found, providing solid evidence for the safety of VC-02 (78). Both studies showed the ability of PEC-01 to mature into insulin-secreting β-cells that enhance the concentration of C-peptide. However, participants did not achieve significant clinical benefits during the trial (79) or the connection between the improved insulin independence and the implanted grafts is unclear (78). It is suggested that by transplanting a larger number of PECs, enriching for the desired cell types, or improving cell survival, patients with T1DM can possibly have better clinical outcomes. It is also worth noting that ViaCyte launched its first clinical trial in 2014 involving VC-01, which includes an encapsulation device that inhibits exposure from immunocytes (82)(NCT02239354). Though the final results have not been released, some preliminary data has been published that reported the safety and efficacy of VC-01.

The two trials have provided substantial proof for translating the theory of PSCs-derived pancreatic islets into clinical application. Vertex, on the other hand, embarked on a new clinical trial from a different perspective, adopting the approach of injecting organoids generated from allogenic stem cells (termed as VX-880) into the portal vein (NCT04786262). VX-880, distinguished from VC-02, is fully differentiated islet-like organoids that possess the potential to restore normoglycemia which has been tested in animal models. Immunosuppression is also compulsory to ensure cell survival within VX-880 (83). These clinical trials bring hopes to millions of patients with T1DM. However, more is needed to employ stem cell-based therapy to exempt them from suffering.

## Limitations and strategies

Currently, tremendous endeavors have been made to recapitulate the development of the pancreas and generate β-cell clusters or pancreatic islet organoids that show significant similarities to native pancreatic islets in terms of morphology, functionality, and global gene expression profiles. Nonetheless, various issues remain, limiting the clinical applications of stem cell-derived pancreatic β-cells. See **Table 3** for a summary of the limitations and their possible strategies.

**Table 3:** Limitations and strategies for stem cells therapy in diabetes.

Limitations	Target	Strategies
Immaturity and heterogeneity	Maturation state	Microenvironment and ECM
		Transplantation of cell clusters *in vivo*
	Sites of transplantation	A more ideal site of transplantation
	Inappropriate heterogeneity	Sorting skills to purify the generated cells
		Encoding suicide genes
Poor vascularization of the grafts	Highly vascularized reliability	Incorporation of amniotic epithelial cells
		Incorporation of HUCPVCs
		Growth factors,such as AEGF-A
Immune rejection	Side-effects and low efficiency of Immunosuppressive drugs	Immunotherapy
		Encapsulation devices
Safety concerns and tumorigenicity	Insertional mutagenesis	Excision systems
		Reprogramming
	Risk of tumorigenicity	Enrichment for the desired cells

### Immaturity and heterogeneity

Early studies mainly focused on producing pure β-cells, which ideally have the capacity to secrete sufficient insulin stimulated by glucose and ameliorate hyperglycemia. Microenvironment and ECM were then found to play a critical role in generating fully mature β-cells, necessitating the generation of islet organoids that contain all types of cells and exhibit better GSIS (72). Disappointingly, despite the closer resemblance to native β-cells discovered by scRNA-seq, some immature cells, and cells that cannot be found in human pancreatic islets, such as SC-EC cells (18), exist in the final product. Thus, further maturation *in vivo* is indispensable, entailing transplanting cell clusters at the final stage of the differentiation protocols into animal models. ScRNA-seq has proved that the grafted islet organoids display better GSIS after transplanting into mice (64), but the underlying cues from the local microenvironment and sophisticated mechanisms that promote β-cell maturation are not fully elucidated. The available alternatives of the sites of transplantation include portal infusion, subcutaneous, intramuscular, and omental/intraperitoneal sites. The limited amount of tissue that can be infused and the impracticability of retrieving islets that have been delivered, considering the possibility of forming teratoma, have restricted the clinical applications of portal fusion (84, 85). A more ideal site of transplantation is in need. Notably, the long duration of maturation cannot be neglected either. In a recent study, it takes 6 months for the grafts to mature.

Of note, subtypes of β-cells in human pancreatic islets of Langerhans were identified according to their differential expression levels of ST8SIA1 and CD9 (86), or Fltp, a marker gene (87). These subtypes co-exist in pancreatic islets and vary in the expression profiles and their functional characteristics, such as GSIS. However, the inappropriate heterogeneity, either in β-cell clusters or islet organoids, does not match the scenario in native islets, thereby hindering the development and maturation of β-cells.

Therefore, it is imperative to purify the generated cell clusters. In addition to improving and simplifying the protocols to generate sufficient supplies of β-cells, sorting skills that can filtrate undesirable cell types and immature cells and enrich the β-cell population are one of the strategies utilized to promote efficiency. CD49a, a positive cell surface marker, and CD9, a negative marker, were identified by scRNA-seq in two studies, which showed that CD49a^+^ CD9^-^ cells displayed better GSIS (18, 65). Encoding suicide genes into the genome of PSCs is another direction of eliminating pluripotent cells or improperly differentiated cells in the final product (88, 89).

### Poor vascularization of the grafts

Disappointingly, although the generation of β-cell products has achieved tremendous success in recent years, it remains a primary hurdle to transplant the final product into patients while ensuring high survival rates simultaneously, which has hindered the clinical use of stem cell-based therapy. As a highly vascularized organ, β-cells rely strongly on capillaries to gain nutrients to survive and modulate blood glucose concentration (90). Thus, their sensitivity to hypoxia necessitates timely vascularization after transplantation. Methods aimed at overcoming this issue include co-transplantation with the parathyroid gland, incorporation of amniotic epithelial cells or human umbilical cord perivascular mesenchymal stromal cells (HUCPVCs) into islet organoids, and the use of growth factors, such as VEGF-A (91–94). Furthermore, when prevascularized with a catheter and transplanted with ∼500 pancreatic islets, the diabetic mice were found to retain euglycemia for >100 days (95).

### Immune rejection

Upon transplantation, the protection of β-cells from immune cells must be assured to achieve satisfying results. Immunosuppressive drugs are needed to prevent allo-rejection for life, but the severe side effects and low efficiency limit their clinical use. Currently, the fast development of immunotherapy has opened a new possibility for tackling autoimmune attacks. Regulatory T-cell therapy is considered a promising strategy that enhances graft survival rates (96, 97). The emergence of CRISPR-Cas9 also revolutionizes the field by ablating HLA-A/-B/-C and HLA class II in PSCs to generate immune tolerant β-cells (98). Notably, upregulation of the immune checkpoint protein PD-L1 also provides protection for the grafts from immune attack (19).

Moreover, encapsulation devices, which provide maximum immune protection with minimal impact on cell viability and function, are also a possible way to solve this issue. Encapsulation devices are used in the study of many diseases, such as cancer, kidney failure, and cartilage defects (99). The ideal cell dispensing device is like a reassembled Langerhans islet that is safe, does not interfere with normal cell physiology, has an optimal nutrient exchange and disposal of cellular waste and facilitates implantation into the recipient and recycling, and, more importantly, protects the cells therein from inflammation and immune system attack (100). The two most essential aspects in the research and fabrication of dispensing devices are physical and chemical properties. Physical properties include the size and shape of the biomaterial used in the dispensing device, material porosity, and surface mechanical properties. Chemical properties include the chemical composition of the biomaterial, the purity of the materials and the interactions between the materials, and the transport and binding capacity of different ions (101). According to their scale, there are mainly two types of encapsulation devices, including microencapsulation and macroencapsulation. Although microcapsules can maximize nutrition and oxygen usage to enhance cell survival since each islet is contained in one capsule, the difficulty of controlling membrane thickness and pore size, as well as the enormous number of microcapsules required for transplantation at one time, limits the application of microencapsulation. On the contrary, by sacrificing the efficiency of transporting soluble factors and metabolites, macroencapsulation can be better harnessed to facilitate the best clinical results (102). Macroencapsulation devices have been employed in clinical trials (NCT02239354, NCT03163511), in which PECs were wrapped by devices that prevent immune attacks and abandon the use of immunosuppressants and devices that allow direct vascular growth, respectively (79, 103, 104). However, fibrosis is observed around the implantation sites due to allogenic responses, which inhibits material exchange and cell survival. Hence, nanofibrous devices are devised to prevent the overgrowth of fibrous tissue that are safe, retrievable, and scalable, holding great promises for the cure of T1DM (105, 106).

### Safety concerns and tumorigenicity

In the process of obtaining iPSCs, retroviruses are the most frequently used vectors to deliver transgenes into the host, which, however, bring about an issue while providing high efficiency. The risk of insertional mutagenesis resulting from the use of retroviruses may disrupt transcriptional profiles of differentiating cells, leading to the production of unwanted cell types or a halt in the differentiation process. Moreover, the pluripotent state also increases the risk of forming teratoma in patients (107). From excision systems aimed at removing transgenes and non-integrative viruses such as Sendai viruses to the use of non-viral methods such as protein, RNA, and chemicals-based reprogramming, scientists have devised a number of strategies to avoid the use of viral vectors to decrease the possibility of teratoma formation and improve safety (108–113). Enrichment for the desired β-cells is also considered to be a promising approach to reducing teratoma.

## Prospects and conclusions

The last decades have witnessed the considerable progress made in the field of pluripotent stem cell-derived β-cell therapy toward the cure for diabetes. Even though β-cells with close resemblance to *bona fide* human β-cells in native islets can be generated with stepwise protocols, barriers that limit their clinical applications still exist. More research is needed to conquer these barriers before cell-based therapy can be fully employed in the treatment of diabetes. To sum up, significant advancements with regard to understanding β-cell differentiation and producing pluripotent stem cell-derived pancreatic β-cells have been revealed, and preliminary data from ongoing PSCs-based clinical trials have confirmed the infinite possibility of this therapy. Although many challenges remain unanswered, we believe that the generation of pluripotent stem cell-derived pancreatic β-cells will represent a promising future for the treatment of diabetes.

## Author contributions

XW and MG collected the literature and wrote the manuscript. YW and YZ wrote, conceived and reviewed the manuscript critically. All authors have read and agreed to the published version of the manuscript.

## Funding

This research was funded by National Natural Science Foundation of China (81702896); Health Research Talent Project of Jilin Province (2020SCZ50); Science and Technology Plan Projects of Jilin Province (20200201539JC); Department of Education of Jilin Province (JJKH20180198KJ); China Scholarship Council (201706175044 and 201606175202); Jilin University Project (2019YX396).

## Conflict of interest

The authors declare that the research was conducted in the absence of any commercial or financial relationships that could be construed as a potential conflict of interest.

## Publisher’s note

All claims expressed in this article are solely those of the authors and do not necessarily represent those of their affiliated organizations, or those of the publisher, the editors and the reviewers. Any product that may be evaluated in this article, or claim that may be made by its manufacturer, is not guaranteed or endorsed by the publisher.
